# Short‐Term Outcomes of Transanal Total Mesorectal Excision for Rectal Cancer: A Multicenter Propensity Score‐Matched Analysis Comparing Patients With and Without Preoperative Neoadjuvant Therapy

**DOI:** 10.1002/ags3.70298

**Published:** 2026-10-14

**Authors:** Takafumi Shima, Hiroki Hamamoto, Takeru Matsuda, Ichiro Takemasa, Keitaro Tanaka, Toshiki Mukai, Jun Watanabe, Sang‐Woong Lee, Seiichiro Yamamoto, Takeshi Naitoh, Ta‐Ta‐Mi Study Group Collaborators from the Japan Society of Laparoscopic Colorectal Surgery, Akihiro Kondo, Akinobu Taketomi, Akiyoshi Kanazawa, Atsushi Ogura, Chikayoshi Tani, Gunpei Yoshimatsu, Hideki Endo, Hideya Kashihara, Hirokazu Suwa, Junichiro Kawamura, Kay Uehara, Kei Kimura, Keiichi Okano, Keiichiro Okuyama, Kenji Okita, Kiyoshi Maeda, Kohei Shigeta, Koji Okabayashi, Koya Hida, Masafumi Inomata, Masahiko Watanabe, Masaji Tani, Masataka Ikeda, Masatsune Shibutani, Masayoshi Iwamoto, Masayuki Ishii, Nobuki Ichikawa, Shigenori Honma, Shinichiro Mori, Shiro Terai, Suguru Hasegawa, Takeshi Okabayashi, Takuya Tokunaga, Tatsunari Fukuoka, Tatsuya Manabe, Tatsuya Shonaka, Tomonori Akagi, Toru Kuramoto, Toru Miyake, Toshikatsu Nitta, Toshiki Mukai, Yoshihito Ide, Yosuke Fukunaga, Yuki Murata, Yukinari Tokoro, Yukitoshi Todate, Yusaku Shogen

**Affiliations:** ^1^ Department of General and Gastroenterological Surgery Osaka Medical and Pharmaceutical University Takatsuki‐shi Osaka Japan; ^2^ Division of Gastrointestinal Surgery, Department of Surgery Kobe University Graduate School of Medicine Kobe Japan; ^3^ Department of Gastroenterological Surgery, Osaka International Medical and Science Center Osaka Keisatsu Hospital Osaka Japan; ^4^ Department of General, Breast and Digestive Surgery Otsu City Hospital Otsu Shiga Japan; ^5^ Department of Colorectal Surgery Cancer Institute Hospital of Japanese Foundation for Cancer Research Tokyo Japan; ^6^ Department of Colorectal Surgery Kansai Medical University Hirakata Japan; ^7^ Department of Gastroenterological Surgery Tokai University School of Medicine Isehara Japan; ^8^ Department of Lower Gastrointestinal Surgery Kitasato University School of Medicine Sagamihara Japan

**Keywords:** neoadjuvant therapy, postoperative complications, propensity score, rectal neoplasms, treatment outcome

## Abstract

**Aim:**

To evaluate the association between preoperative neoadjuvant therapy and short‐term outcomes of transanal total mesorectal excision for rectal cancer.

**Methods:**

We retrospectively analyzed patients who underwent transanal total mesorectal excision at 26 Japanese institutions between 2012 and 2019. Patients who received neoadjuvant therapy were matched 1:1 with patients who did not. Perioperative, postoperative, and pathological outcomes were compared.

**Results:**

After matching, 294 patients (147 per group) were analyzed. Operative time was longer in the neoadjuvant therapy group than in the non‐neoadjuvant therapy group (median, 510.0 vs. 382.0 min), with an adjusted mean difference of 129.25 min (95% CI, 87.90–170.59; *p* < 0.001). Clavien–Dindo grade ≥ III complications occurred in 18/147 patients (12.2%) without neoadjuvant therapy and 16/147 (10.9%) with neoadjuvant therapy (adjusted OR, 0.90; 95% CI, 0.44–1.85; *p* = 0.769). Full‐cohort conventional and Firth logistic analyses and marginal standardization likewise did not identify a statistically significant association. Neoadjuvant therapy remained associated with longer operative time after adjustment for operative procedure, lateral pelvic lymph node dissection, and abdominal approach. Resection‐margin involvement did not differ significantly between groups.

**Conclusion:**

Neoadjuvant therapy was associated with longer operative time during transanal total mesorectal excision, whereas no statistically significant association with severe postoperative morbidity was detected. Sensitivity analyses likewise did not identify a statistically significant association, supporting the technical feasibility of transanal total mesorectal excision after neoadjuvant therapy in experienced centers.

## Abstract 2

In this multicenter propensity score‐matched study, neoadjuvant therapy was associated with longer operative time during trans‐anal total mesorectal excision for rectal cancer. However, no statistically significant association was observed between neoadjuvant therapy and Clavien–Dindo grade ≥ III postoperative complications, supporting the technical feasibility of TaTME after neoadjuvant therapy in experienced centers.

**Abbreviations**
APR: abdominoperineal resection
ASA‐PS: American Society of Anesthesiologists physical status
BMI: body mass index
CA19‐9: carbohydrate antigen 19–9
CD: Clavien–Dindo
CEA: carcinoembryonic antigen
CI: confidence interval
CRT: chemoradiotherapy
IQR: interquartile range
ISR: intersphincteric resection
LAR: low anterior resection
LLND: lateral pelvic lymph node dissection
LUTD: lower urinary tract dysfunction
NAC: neoadjuvant chemotherapy
NAT: neoadjuvant therapy
OR: odds ratio
pCR: pathological complete response
PS: propensity score
SMD: standardized mean difference
SSI: surgical‐site infection
TaTME: transanal total mesorectal excision
TME: total mesorectal excision
TNT: total neoadjuvant therapy
TPE: total pelvic exenteration

## 1 Introduction

Total mesorectal excision (TME) is crucial for achieving local control of tumors in the mid‐ and lower rectum, and transanal TME (TaTME) was developed to overcome the technical challenges of laparoscopic pelvic dissection for rectal cancer, particularly in patients who are male or obese or who have a narrow pelvis or mid‐ to low‐rectal tumors [1, 2, 3]. Its improved visualization and maneuverability may facilitate precise dissection in the deep pelvis.

The treatment options for locally advanced rectal cancer include neoadjuvant chemotherapy (NAC) and neoadjuvant chemoradiotherapy (NACRT); in recent years, total neoadjuvant therapy (TNT) has been adopted to increase the pathological complete response (pCR) rate and improve systemic disease control [4, 5, 6]. As the use of these neoadjuvant treatment modalities expands, understanding their influence on the technical feasibility, perioperative safety, and pathological outcomes of TaTME has become increasingly important.

A network meta‐analysis of 29 randomized controlled trials involving 6237 patients found broadly comparable perioperative and oncological outcomes among open, laparoscopic, robotic, and transanal approaches. Although laparoscopic surgery was associated with a higher risk of circumferential resection‐margin involvement than transanal surgery, this analysis compared surgical approaches and did not specifically evaluate the influence of NAT on TaTME outcomes [7]. In addition, a retrospective comparative study reported comparable short‐term outcomes between TaTME and robotic TME in patients with low rectal cancer following neoadjuvant chemoradiotherapy. However, its retrospective design and single‐centre setting limit the generalizability of those findings [8]. Nevertheless, evidence regarding the effects of NAT on the short‐term outcomes of TaTME remains limited, particularly in multicentre populations.

In the parent nationwide Ta‐Ta‐Mi study, 706 patients who underwent TaTME at 26 Japanese institutions were evaluated. The 3‐year cumulative local recurrence rate was 6.82%, and oncological outcomes were considered acceptable [9]. However, that analysis focused primarily on local recurrence and did not specifically assess the influence of NAT on short‐term surgical outcomes. Therefore, in this study, we evaluated the impact of NAT on the short‐term perioperative, pathological, and complication outcomes of TaTME for rectal cancer using a propensity score‐matched cohort derived from 26 institutions.

## 2 Patients and Methods

### 2.1 Study Design and Reporting

This retrospective multicenter cohort study was conducted in accordance with the STROBE statement [10] for observational research and was conducted across 26 institutions affiliated with the Japan Society of Laparoscopic Colorectal Surgery. Patients diagnosed with primary rectal cancer who underwent TaTME at one of the participating institutions between January 2012 and December 2019 were included. The inclusion criteria were histologically diagnosed adenocarcinoma or adenosquamous carcinoma with the distal edge of the tumor located within 12 cm of the anal verge. Patients with recurrent disease, multiple cancers, or ulcerative colitis and those who underwent concomitant surgery for another disease were excluded. Hartmann's procedure or total pelvic exenteration were excluded a priori (Figure 1). The choice of NAT regimen was not standardized by the study protocol and was determined according to clinical practice at each participating institution; detailed institution‐specific eligibility criteria and the interval between completion of NAT and surgery were not prospectively collected. Similarly, criteria for no, unilateral, or bilateral LLND were not standardized across institutions. Tumor height was collected in the parent Ta‐Ta‐Mi study, in which the median distance from the anal verge was 4 cm, and has been previously reported [9], whereas systematic MRI‐based assessments of mesorectal fascia involvement and extramural venous invasion were not included in the present database. Detailed information regarding sphincter or levator ani muscle invasion, the preoperatively planned surgical procedure, and institution‐specific eligibility criteria for ISR or other sphincter‐preserving procedures was not prospectively collected. CRM involvement was defined as a tumor‐to‐circumferential resection margin distance of ≤ 1 mm, and a distance > 1 mm was classified as CRM negative. Pathological assessment was performed locally at each institution without centralized pathological review, and detailed specimen‐processing and CRM measurement procedures were not standardized by the study protocol.

**FIGURE 1:**
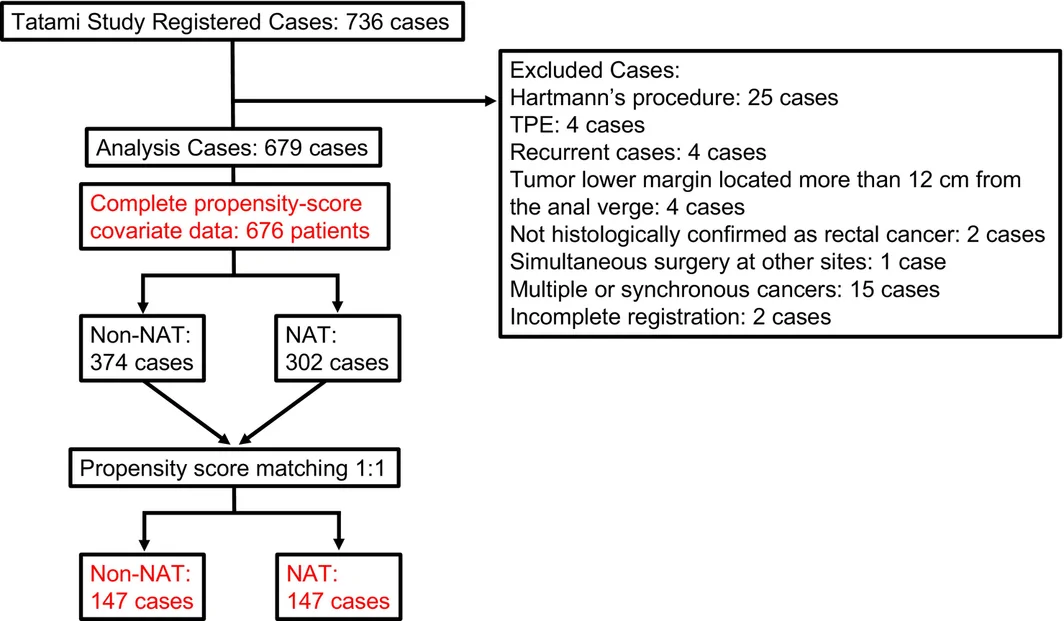
Study flow diagram. NAT, neoadjuvant therapy; TPE, total pelvic exenteration.

The study was approved by the Institutional Review Board and Ethics Committee of Kobe University Graduate School of Medicine (IRB reference code: B220092). The need for informed consent was waived in view of the anonymity of the study data. However, in accordance with the Japanese ethical regulations, Japanese Personal Information Protection Law, and instructions of the Ethics Committees of each institution, consent was secured via the opt‐out route. Information regarding the purpose and methodology of the study was disclosed on the hospital noticeboard and/or website and opportunities to opt out were provided to the extent possible.

### 2.2 Endpoints

The pre‐specified primary endpoint was postoperative complications of Clavien–Dindo (CD) grade ≥ III, classified according to the original CD classification [11]. Secondary endpoints included (i) CD grade ≥ II complications; (ii) specific complications—anastomotic leakage, postoperative ileus, postoperative bleeding, lower urinary tract dysfunction (LUTD), and surgical‐site infection (SSI); (iii) operative time, intraoperative blood loss, number of harvested lymph nodes, and length of hospital stay; and (iv) pathological findings (resection margins, pT, pN, pM, lymphatic invasion, vascular invasion, perineural invasion, tumor budding, and lateral pelvic lymph‐node metastasis).

### 2.3 Statistical Analysis

Categorical variables are presented as numbers and percentages and were compared using the chi‐square test or Fisher's exact test, as appropriate. Continuous variables are presented as median and interquartile range (IQR) and were compared using the Mann–Whitney U test.

To reduce treatment selection bias and potential confounding, propensity score (PS) matching was performed using the MatchIt package [12, 13]. Patients in the neoadjuvant therapy group were matched 1:1 to those in the non‐neoadjuvant therapy group using nearest‐neighbor matching without replacement with a caliper width of 0.2 standard deviations of the logit of the propensity score [12]. Age, sex, body mass index, American Society of Anesthesiologists physical status, carcinoembryonic antigen (CEA), carbohydrate antigen 19–9 (CA19‐9), previous abdominal surgery, and clinical tumor stage (cT, cN, and cM; TNM classification of the 8th edition of the American Joint Committee on Cancer) were included in the propensity score model. Exact matching was imposed on categorized age (≥ 70 vs. < 70 years), BMI (≥ 25 vs. < 25 kg/m^2^), ASA‐PS (1–2 vs. 3–4), CEA (≥ 5 vs. < 5 ng/mL), CA19‐9 (≥ 37 vs. < 37 U/mL), cT (0–3 vs. 4), and cN. After matching, covariate balance was assessed using standardized mean differences (SMDs); an SMD < 0.1 was considered indicative of good balance.

After matching, continuous BMI showed only minor residual imbalance and was therefore additionally adjusted for in the matched outcome models. Continuous outcomes were analyzed using weighted linear regression and binary outcomes using weighted logistic regression with cluster‐robust standard errors by matched subclass. For sparse binary outcomes, absolute risk differences were additionally estimated using weighted linear probability models with matched‐subclass cluster‐robust standard errors; risk differences are reported as NAT minus non‐NAT in percentage points. For the primary endpoint, sensitivity analyses included conventional multivariable logistic regression in the complete‐case full cohort with institution‐cluster robust standard errors, Firth logistic regression, and marginal standardization. Additional analyses evaluated NAT according to specific regimen (NAC, CRT, TNT, and radiotherapy alone), a post‐matching operative‐time model adjusted for BMI, operative procedure, LLND, and abdominal approach, a no‐LLND subgroup analysis, and NAT × LLND interaction testing. Anastomotic leakage was additionally analyzed among patients undergoing LAR or ISR, with adjustment for BMI and diverting stoma. All tests were two‐sided, and *p* < 0.05 was considered statistically significant.

## 3 Results

### 3.1 Patient Characteristics

A total of 679 patients with rectal cancer who underwent TaTME at the 26 participating institutions were eligible for inclusion. Complete propensity‐score covariate data were available for 676 patients. After 1:1 propensity score matching, 294 patients (147 per group) were included in the matched analysis (Table 1). After matching, good balance was achieved for the prespecified covariates, except for minor residual imbalance in continuous BMI (absolute SMD = 0.113; Table 1). Continuous BMI was therefore additionally adjusted for in the matched outcome models.

**TABLE 1:** Patient characteristics.

Factor	Category	Before matching			After matching		
		Non‐neoadjuvant therapies group (*N* = 375)	Neoadjuvant therapies group (*N* = 304)	SMD	Non‐neoadjuvant therapies group (*N* = 147)	Neoadjuvant therapies group (*N* = 147)	SMD
		*n* (%)	*n* (%)		*n* (%)	*n* (%)	
Age (years)	< 70	225 (60.0)	218 (71.7)	−0.260	104 (70.7)	104 (70.7)	0.097
	≥ 70	150 (40.0)	86 (28.3)		43 (29.3)	43 (29.3)	
Sex	Male	259 (69.1)	211 (69.4)	−0.007	103 (70.1)	101 (68.7)	0.030
	Female	116 (30.9)	93 (30.6)		44 (29.9)	46 (31.3)	
BMI (kg/m^2^)	< 25	268 (71.5)	221 (72.7)	−0.028	109 (74.1)	109 (74.1)	0.113
	≥ 25	107 (28.5)	83 (27.3)		38 (25.9)	38 (25.9)	
ASA‐PS	I, II	340 (90.7)	289 (95.1)	−0.203	145 (98.6)	145 (98.6)	0.000
	III, IV	35 (9.3)	15 (4.9)		2 (1.4)	2 (1.4)	
CEA (ng/mL)	< 5	273 (72.8)	162 (53.3)	0.391	99 (67.3)	99 (67.3)	0.012
	≥ 5	102 (27.2)	142 (46.7)		48 (32.7)	48 (32.7)	
CA19‐9 (U/mL)	< 37	345 (92.0)	262 (86.2)	0.169	141 (95.9)	141 (95.9)	0.010
	≥ 37	30 (8.0)	42 (13.8)		6 (4.1)	6 (4.1)	
Previous abdominal surgery	−	303 (80.8)	238 (78.3)	0.061	115 (78.2)	115 (78.2)	0.000
	+	72 (19.2)	66 (21.7)		32 (21.8)	32 (21.8)	
cT	0–3	333 (89.0)	221 (73.2)	0.414	125 (85.0)	125 (85.0)	0.000
	4	41 (11.0)	81 (26.8)		22 (15.0)	22 (15.0)	
cN	0	262 (69.9)	106 (34.9)	0.734	76 (51.7)	76 (51.7)	0.000
	≥ 1	113 (30.1)	198 (65.1)		71 (48.3)	71 (48.3)	
cM	0	359 (95.7)	254 (83.6)	0.329	135 (91.8)	135 (91.8)	0.000
	1	16 (4.3)	50 (16.4)		12 (8.2)	12 (8.2)	

*Note:* Clinical T category was unavailable in one non‐NAT patient and two NAT patients before matching; these patients were excluded from the propensity‐score analysis. Age, BMI, CEA, and CA19‐9 are presented as categories, their SMDs were calculated using continuous values.

Abbreviations: ASA‐PS, American Society of Anesthesiologists physical status; BMI, body mass index; CA19‐9, carbohydrate antigen 19–9; CEA, carcinoembryonic antigen; NAT, neoadjuvant therapy; SMD, standardized mean difference.

### 3.2 Perioperative Outcomes

Perioperative outcomes are shown in Tables 2 and 3. After matching, the overall distribution of operative procedure no longer differed significantly between groups, and diverting stoma use was also similar (94/147 (63.9%) in the non‐NAT group vs. 102/147 (69.4%) in the NAT group; *p* = 0.387). Differences in LLND and abdominal approach remained clinically relevant and were therefore examined in additional sensitivity analyses rather than being incorporated into the propensity score, because these procedural variables may occur downstream of NAT and treatment response.

**TABLE 2:** Perioperative surgical procedures after propensity score matching.

Variable	Category	Non‐NAT (*N* = 147), *n* (%)	NAT (*N* = 147), *n* (%)	*p*
Operative procedure	LAR	38 (25.9%)	31 (21.1%)	0.314
	ISR	73 (49.7%)	86 (58.5%)	
	APR	36 (24.5%)	30 (20.4%)	
Abdominal approach	Laparoscopic	127 (86.4%)	91 (61.9%)	< 0.001
	Robotic	19 (12.9%)	49 (33.3%)	
	Open	1 (0.7%)	7 (4.8%)	
Diverting stoma	−	53 (36.1%)	45 (30.6%)	0.387
	+	94 (63.9%)	102 (69.4%)	
Lymph node dissection	D2	14 (9.5%)	4 (2.7%)	0.029
	D3	133 (90.5%)	143 (97.3%)	
LLND	−	105 (71.4%)	67 (45.6%)	< 0.001
	Unilateral	11 (7.5%)	16 (10.9%)	
	Bilateral	31 (21.1%)	64 (43.5%)	
Autonomic nerve resection	−	141 (95.9%)	133 (90.5%)	0.079
	Unilateral	5 (3.4%)	7 (4.8%)	
	Bilateral	1 (0.7%)	7 (4.8%)	
Combined resection of other organs	−	138 (93.9%)	135 (91.8%)	0.651
	+	9 (6.1%)	12 (8.2%)	
Transanal approach teams	One‐team	34 (23.1%)	52 (35.4%)	0.029
	Two‐team	113 (76.9%)	95 (64.6%)	
Transanal approach grade	I	34 (23.1%)	35 (23.8%)	0.651
	II	101 (68.7%)	104 (70.7%)	
	III	12 (8.2%)	8 (5.4%)	

*Note:* *p* values compare the distribution of each procedural characteristic between matched groups; Pearson chi‐square tests were used for multi‐category variables and continuity‐corrected/fisher‐type 2 × 2 comparisons for binary variables.

Abbreviations: APR, abdominoperineal resection; ISR, intersphincteric resection; LAR, low anterior resection; LLND, lateral pelvic lymph node dissection; NAT, neoadjuvant therapy.

**TABLE 3:** Perioperative quantitative outcomes after propensity score matching.

Outcome	Non‐NAT (*N* = 147) median (IQR)	NAT (*N* = 147) median (IQR)	BMI‐adjusted mean difference	95% CI	*p*
Operative time (min)	382.0 (289.5–474.0)	510.0 [374.5–667.0]	129.25	87.90 to 170.59	< 0.001
Intraoperative blood loss (g)	50 (20–120)	40 [10–112]	25.44	−44.99 to 95.88	0.479
Number of lymph nodes dissected	17 (12–29)	22 [13–38]	3.09	−0.26 to 6.44	0.071
Length of hospital stay (days)	17 (14–23)	18 [14–24]	−0.11	−3.90 to 3.68	0.954

Abbreviations: CI, confidence interval; IQR, interquartile range; NAT, neoadjuvant therapy.

Operative time was longer in the NAT group than in the non‐NAT group (median, 510.0 min (IQR, 374.5–667.0) vs. 382.0 min (IQR, 289.5–474.0), respectively), with an adjusted mean difference of 129.25 min (95% CI, 87.90–170.59; *p* < 0.001) (Table 3). In the subgroup of patients who did not undergo LLND, NAT remained associated with longer operative time than non‐NAT, with an adjusted 13.9% increase in operative time (95% CI, 1.4%–27.9%; *p* = 0.029; Table 4). There was no significant interaction between NAT and LLND extent (global P for interaction = 0.715; Table 5). In a post‐matching sensitivity model additionally adjusting for BMI, operative procedure, LLND, and abdominal approach, NAT remained associated with a 14.0% longer operative time (95% CI, 5.3%–23.4%; *p* = 0.001; Table S1).

**TABLE 4:** Operative time in patients without lateral lymph node dissection.

Outcome	Non‐NAT (*N* = 105)	NAT (*N* = 67)	Adjusted % change	95% CI	*p*
Operative time, min, median (IQR)	335 (266–422)	382 (296–491)	13.9	1.4 to 27.9	0.029

*Note:* Estimates were derived from an analysis restricted to patients who did not undergo LLND.

Abbreviations: CI, confidence interval; IQR, interquartile range; NAT, neoadjuvant therapy.

**TABLE 5:** NAT × LLND interaction analysis.

Term	Comparison/interpretation	Adjusted % change	95% CI	*p*	Role
NAT	NAT vs. Non‐NAT within No‐LLND	14.2	1.6–28.4	0.027	Main effect
Unilateral LLND	Unilateral LLND vs. No LLND within Non‐NAT	35.5	15.9–58.3	0.000	Main effect
Bilateral LLND	Bilateral LLND vs. No LLND within Non‐NAT	52.5	37.5–69.3	0.000	Main effect
NAT × unilateral LLND	Modification of NAT association with unilateral LLND	4.3	−13.6–26.1	0.660	Interaction
NAT × bilateral LLND	Modification of NAT association with bilateral LLND	6.6	−8.6–24.4	0.417	Interaction
Global NAT × LLND interaction test				*p* for interaction = 0.715	

*Note:* Estimates were derived from the interaction model including NAT, LLND extent, and NAT × LLND interaction terms.

Abbreviations: CI, confidence interval; IQR, interquartile range; LLND, lateral pelvic lymph node dissection; NAT, neoadjuvant therapy.

For the primary endpoint, CD grade ≥ III complications occurred in 18 of 147 patients (12.2%) in the non‐NAT group and 16 of 147 patients (10.9%) in the NAT group (BMI‐adjusted matched‐pair OR, 0.90; 95% CI, 0.44–1.85; *p* = 0.769) (Table 6). Among patients undergoing LAR or ISR, anastomotic leakage occurred in 13 of 111 patients (11.7%) in the non‐NAT group and 4 of 117 patients (3.4%) in the NAT group. In the complete‐case full cohort (*n* = 676), the adjusted OR was 1.16 (95% CI, 0.67–2.03; *p* = 0.595) using conventional multivariable logistic regression with institution‐cluster robust standard errors and 1.16 (95% CI, 0.70–1.94; *p* = 0.562) using Firth logistic regression. Marginal standardization yielded adjusted risks of 11.5% for non‐NAT and 13.1% for NAT, corresponding to an adjusted risk difference of +1.59 percentage points (95% CI, −4.46 to +7.64; *p* = 0.606; Table S2).

**TABLE 6:** Postoperative complication outcomes after propensity score matching.

Outcome	Non‐NAT, *n*/*N* (%)	NAT, *n*/*N* (%)	Measure	Adjusted estimate (95% CI)	*p*
CD grade ≥ II complications	54/147 (36.7%)	48/147 (32.7%)	OR	0.86 (0.52–1.44)	0.571
CD grade ≥ III complications	18/147 (12.2%)	16/147 (10.9%)	OR	0.90 (0.44–1.85)	0.769
Anastomotic leakage^a^	13/111 (11.7%)	4/117 (3.4%)	OR	0.28 (0.09–0.88)	0.029
Ileus	12/147 (8.2%)	12/147 (8.2%)	OR	1.08 (0.45–2.56)	0.866
Postoperative bleeding	1/147 (0.7%)	1/147 (0.7%)	RD, pp	−0.01 (−1.88 to 1.86)	0.993
LUTD	7/147 (4.8%)	7/147 (4.8%)	OR	0.99 (0.32–3.09)	0.991
Surgical site infection	7/147 (4.8%)	6/147 (4.1%)	RD, pp	−0.90 (−5.78 to 3.99)	0.719

*Note:* For sparse outcomes, adjusted risk differences were estimated using weighted linear probability models with matched‐subclass cluster‐robust standard errors. RD is expressed as NAT minus non‐NAT in percentage points.

Abbreviations: CD, Clavien–Dindo; CI, confidence interval; ISR, intersphincteric resection; LAR, low anterior resection; LUTD, lower urinary tract dysfunction; NAT, neoadjuvant therapy; OR, odds ratio; pp., percentage points; RD, risk difference.

^a^ Anastomotic leakage was analyzed only among patients undergoing LAR or ISR and was adjusted for BMI and diverting stoma.

In the exploratory multivariable analysis of the complete‐case full cohort (*n* = 676), NAT remained associated with longer operative time after adjustment for demographic, clinicopathological, and operative variables, corresponding to an 8.3% increase in operative time (95% CI, 2.3%–14.7%; *p* = 0.006). Male sex, higher BMI, previous abdominal surgery, cT2–4 disease compared with cT0, LLND, APR, and an open surgical approach were also associated with longer operative time, whereas the two‐team transanal approach was associated with shorter operative time (Table 7).

**TABLE 7:** Main multivariable analysis of factors associated with operative time.

Variable	Comparison/coding	Approximate change in operative time (%)	95% CI	*p*
Neoadjuvant therapy	Non‐NAT	Reference	—	—
	NAT	+8.3	2.3–14.7	0.006
Sex	Male	Reference	—	—
	Female	−7.4	−12.0 to −2.7	0.003
BMI	Per 1‐kg/m^2^ increase	+1.3	0.7–1.9	< 0.001
ASA‐PS	I, II	Reference	—	—
	III, IV	−6.4	−14.4 to 2.4	0.147
CEA	log (*x* + 1)	+0.8	−1.9 to 3.5	0.578
CA19‐9	log (*x* + 1)	+2.3	−0.1 to 4.6	0.058
Previous abdominal surgery	−	Reference	—	—
	+	+6.0	0.3–12.0	0.038
cT	0	Reference	—	—
	1	+12.9	−2.9 to 31.4	0.115
	2	+16.8	1.2–34.9	0.034
	3	+21.1	5.9–38.5	0.005
	4	+29.0	10.8–50.2	0.001
cN	−	Reference	—	—
	+	+1.5	−4.0 to 7.4	0.599
cM	−	Reference	—	—
	+	+5.0	−3.1 to 13.8	0.236
LLND	−	Reference	—	—
	Unilateral	+24.3	14.8–34.7	< 0.001
	Bilateral	+54.6	45.7–64.1	< 0.001
Operative procedure	LAR	Reference	—	—
	ISR	+2.7	−3.4 to 9.3	0.392
	APR	+7.9	1.4–14.8	0.017
Surgical approach	Open	Reference	—	—
	Minimally invasive surgery^a^	−16.6	−35.8 to −0.1	0.049
Transanal approach teams	One‐team	Reference	—	—
	Two‐team	−17.6	−21.6 to −13.4	< 0.001^a^

Abbreviations: APR, abdominoperineal resection; ASA‐PS, American Society of Anesthesiologists physical status; BMI, body mass index; CA19‐9, carbohydrate antigen 19–9; CEA, carcinoembryonic antigen; CI, confidence interval; ISR, intersphincteric resection; LAR, low anterior resection; LLND, lateral lymph node dissection; NAT, neoadjuvant therapy.

^a^ Minimally invasive surgery includes laparoscopic and robotic approaches.

In the regimen‐specific analysis, operative time, blood loss, and postoperative complications were compared among the NAC, CRT, TNT, and radiotherapy‐alone groups. CD grade ≥ III complications occurred in 20/180 (11.1%), 17/102 (16.7%), 3/12 (25.0%), and 2/8 (25.0%) patients receiving NAC, CRT, TNT, and radiotherapy alone, respectively, without a statistically significant overall difference (*p* = 0.164; Table S3). Operative time was longest in the radiotherapy‐alone group. However, when NAC was used as the reference in the adjusted analysis, operative time did not differ significantly among CRT, TNT, and radiotherapy alone (Table S4).

### 3.3 Pathological Findings

Pathological findings are summarized in Table 8. Distal margin positivity was rare, occurring in 1 of 146 evaluable patients (0.7%) in the non‐NAT group and 1 of 147 patients (0.7%) in the NAT group. The NAT group had significantly lower frequencies of lymphatic invasion (23.8% vs. 43.5%; adjusted OR, 0.41; 95% CI, 0.24–0.69; *p* < 0.001) and venous invasion (38.8% vs. 64.6%; adjusted OR, 0.34; 95% CI, 0.20–0.56; *p* < 0.001) than the non‐NAT group. In contrast, no significant between‐group differences were observed in p/ypT category (*p* = 0.607), p/ypN status (*p* = 0.848), perineural invasion (35/147 (23.8%) vs. 27/147 (18.4%); adjusted OR, 0.67; 95% CI, 0.38–1.17; *p* = 0.159), or tumor differentiation (differentiated histology: 138/147 (93.9%) vs. 130/147 (88.4%); adjusted OR, 1.95; 95% CI, 0.83–4.58; *p* = 0.127). Lateral pelvic lymph‐node metastasis was numerically more frequent in the NAT group (16/147 (10.9%) vs. 8/147 (5.4%); adjusted OR, 2.06; 95% CI, 0.92–4.63; *p* = 0.079), although the difference did not reach statistical significance.

**TABLE 8:** Pathological findings after propensity score matching.

Variable	Category	Non‐NAT (*N* = 147), *n* (%)	NAT (*N* = 147), *n* (%)	Adjusted effect	95% CI	*p*
Distal margin	Negative	145 (98.6%)	146 (99.3%)	RD +0.07 pp	−1.64 to 1.78	0.935
	Positive	1 (0.7%)	1 (0.7%)			
	Unknown	1 (0.7%)	0 (0.0%)			
CRM	Negative (> 1 mm)	130 (88.4%)	132 (89.8%)	OR 0.71	0.28 to 1.81	0.472
	Positive (≤ 1 mm)	11 (7.5%)	8 (5.4%)			
	Unknown	6 (4.1%)	7 (4.8%)			
LPN metastasis	Negative	139 (94.6%)	131 (89.1%)	OR 2.06	0.92 to 4.63	0.079
	Positive	8 (5.4%)	16 (10.9%)			
Lymphatic invasion	Negative	83 (56.5%)	112 (76.2%)	OR 0.41	0.24 to 0.69	< 0.001
	Positive	64 (43.5%)	35 (23.8%)			
Venous invasion	Negative	52 (35.4%)	90 (61.2%)	OR 0.34	0.20 to 0.56	< 0.001
	Positive	95 (64.6%)	57 (38.8%)			
Perineural invasion	Negative	93 (63.3%)	108 (73.5%)	OR 0.67	0.38 to 1.17	0.159
	Positive	35 (23.8%)	27 (18.4%)			
	Unknown	19 (12.9%)	12 (8.2%)			
Budding	BD1	58 (39.5%)	74 (50.3%)	—	—	0.006
	BD2	10 (6.8%)	8 (5.4%)			
	BD3	10 (6.8%)	0 (0.0%)			
	Unknown	69 (46.9%)	65 (44.2%)			
p/ypT	0–2/Tis	72 (49.0%)	75 (51.0%)	OR 1.11	0.75 to 1.65	0.607
	3–4	75 (51.0%)	72 (49.0%)			
p/ypN	Negative	98 (66.7%)	100 (68.0%)	OR 0.95	0.58 to 1.56	0.848
	Positive	49 (33.3%)	47 (32.0%)			
Pathological M	Negative	136 (92.5%)	140 (95.2%)	RD −3.05 pp	−6.38 to 0.28	0.074
	Positive	11 (7.5%)	7 (4.8%)			
Tumor differentiation	Differentiated	138 (93.9%)	130 (88.4%)	OR 1.95	0.83 to 4.58	0.127
	Others	9 (6.1%)	17 (11.6%)			

*Note:* For sparse outcomes, adjusted risk differences were estimated using weighted linear probability models with matched‐subclass cluster‐robust standard errors. RD is expressed as NAT minus non‐NAT in percentage points. Percentages are based on the total matched cohort; effect estimates for distal margin and CRM were calculated among patients with evaluable margin data.

Abbreviations: BD, tumor budding grade; CI, confidence interval; CRM, circumferential resection margin; LPN, lateral pelvic lymph node; NAT, neoadjuvant therapy; OR, odds ratio; RD, risk difference.

## 4 Discussion

This propensity‐score‐matched analysis investigated the impact of NAT on short‐term outcomes following TaTME for rectal cancer. The principal finding was that NAT was associated with longer operative time. No statistically significant association between NAT and CD grade ≥ III postoperative complications was detected in the matched analysis. Sensitivity analyses using full‐cohort conventional logistic regression, Firth logistic regression, and marginal standardization likewise did not identify a statistically significant association between NAT and severe postoperative complications. However, effect estimates varied across analyses, and the confidence intervals remained wide; therefore, clinically relevant benefit or harm could not be excluded. These results should not be interpreted as demonstrating equivalence or the complete absence of an effect.

With regard to morbidity, these results align with recent evidence suggesting that NAT, including chemotherapy, chemoradiation, and TNT, does not increase the incidence of serious complications following rectal‐cancer surgery in general [5, 6, 14, 15, 16, 17, 18, 19]. The COLOR III trial reported comparable short‐term morbidity between TaTME and laparoscopic TME, although the trial did not specifically address NAT [20]. Similarly, the Liu et al. randomised comparison of TaTME versus laparoscopic TME likewise documented similar severe morbidity in both arms, again without directly randomising NAT exposure [21]. A 2019 network meta‐analysis pooling open, laparoscopic, robotic, and transanal mesorectal‐excision trials found no significant differences in intra‐operative morbidity, conversion rate, or Grade III/IV complications across approaches [7]. A recent systematic review of NAC alone similarly found no excess in postoperative morbidity compared with NACRT [22]. The overall CD grade ≥ III complication rate in the matched cohort was 11.6% (34/294), which falls within the 10%–15% range reported in previous multicentre trials and supports the external consistency of the present findings.

The longer operative time observed after NAT is compatible with increased operative complexity related to treatment‐associated tissue edema, fibrosis or less distinct anatomical planes, but it may also reflect differences in tumor characteristics, LLND, operative procedure, abdominal approach, institutional practice, and surgeon experience [15, 23, 24]. Importantly, the association persisted in the post‐matching sensitivity model adjusting for BMI, operative procedure, LLND, and abdominal approach. LLND itself was strongly associated with longer surgery, but NAT remained associated with longer operative time even among patients who did not undergo LLND, and no significant NAT × LLND interaction was detected. These findings suggest that LLND and other procedural factors contribute to operative complexity but do not fully explain the observed association between NAT and operative duration. Residual confounding nevertheless remains possible. By contrast, the two‐team transanal approach was independently associated with shorter operative time, consistent with previous reports suggesting greater procedural efficiency with simultaneous abdominal and transanal dissection [25].

Although operative time was longest in the radiotherapy‐alone group in the descriptive analysis, adjusted regimen‐specific comparisons did not identify significant differences among NAT regimens. The indications for NAC, CRT, and TNT were not standardized by the study protocol; detailed institution‐specific selection criteria were not collected, and the interval between completion of NAT and surgery was unavailable. Accordingly, regimen‐specific findings should be considered exploratory.

The pathological findings showed significantly lower rates of lymphatic and venous invasion in the NAT group than in the non‐NAT group (lymphatic invasion, 23.8% vs. 43.5%; venous invasion, 38.8% vs. 64.6%; both *p* < 0.001), consistent with the known treatment‐related pathological response to NAT reported in randomized trials and meta‐analyses [14, 15, 16, 17, 18, 19]. In contrast, no significant between‐group differences were observed in the grouped p/ypT or p/ypN categories. TNT, in particular, has been associated with higher pathological complete response rates [16, 17], although regimen‐specific pathological effects could not be definitively assessed in the present study.

Importantly, no significant difference in resection‐margin involvement was detected. Distal margin positivity was observed in 1 of 146 evaluable patients (0.7%) in the non‐NAT group and 1 of 147 patients (0.7%) in the NAT group (adjusted risk difference, +0.07 percentage points; 95% CI, −1.64 to 1.78; *p* = 0.935). CRM involvement, defined as a margin distance of ≤ 1 mm, was observed in 11 of 141 evaluable patients (7.8%) in the non‐NAT group and 8 of 140 patients (5.7%) in the NAT group (adjusted OR, 0.71; 95% CI, 0.28–1.81; *p* = 0.472). These findings are clinically relevant because fibrosis and tissue edema after NAT may obscure the dissection plane and potentially increase the risk of an inadequate resection margin. Nevertheless, in this multicentre cohort treated at experienced centres, TaTME after NAT was not associated with an increased rate of short‐term pathological margin involvement.

An unexpected finding was that anastomotic leakage was less frequent in the NAT group than in the non‐NAT group among patients undergoing LAR or ISR (3.4% vs. 11.7%, respectively). Several factors may explain this difference. Tumor regression after NAT may facilitate distal rectal transection and pelvic dissection in selected patients, potentially reducing technical difficulty during reconstruction. In addition, differences in patient selection, institutional practice, anastomotic technique, perfusion assessment, and other perioperative management factors that were not captured in the database may have contributed to this finding. Although the analysis was restricted to patients with an anastomosis and adjusted for diverting stoma, the numerically lower leakage rate in the NAT group was observed in both patients with and without a diverting stoma. However, the number of leakage events was small, particularly among patients without a diverting stoma, and the timing of surgery after NAT and detailed technical factors related to anastomosis were unavailable. Therefore, this finding should be regarded as exploratory and should not be interpreted as evidence that NAT itself protects against anastomotic leakage.

Several limitations should be acknowledged. First, this was a retrospective observational study, and assignment to NAT was non‐randomised. Moreover, the NAT group included only patients who ultimately proceeded to TaTME. Patients who developed disease progression, severe treatment toxicity, or another reason for not undergoing surgery were not captured. The findings therefore apply to selected surgical patients and not to the safety or effectiveness of the complete NAT‐to‐surgery treatment strategy. Second, NAT regimen selection, LLND criteria, and sphincter‐preserving surgical indications were not standardized across participating institutions, and the interval between completion of NAT and surgery was not available. In addition, group‐specific tumor height and MRI‐based assessments of mesorectal fascia involvement and extramural venous invasion were unavailable, limiting our ability to fully account for anatomical tumor characteristics. Third, although propensity score matching substantially improved measured baseline balance, residual and unmeasured confounding remains possible. Fourth, pathological assessment was performed locally without centralized review or a standardized detailed CRM processing and measurement protocol. Fifth, several clinically important outcomes were rare, resulting in limited statistical precision despite the use of risk differences and Firth sensitivity analyses. Finally, all participating centers were experienced Japanese institutions, which may limit generalizability. Conversely, the nationwide multicenter design and the availability of detailed clinical, operative, and pathological data represent important strengths. This study therefore provides clinically relevant evidence regarding the impact of NAT on the short‐term outcomes of TaTME.

## 5 Conclusions

In this multicentre retrospective cohort of selected patients who underwent TaTME, NAT was associated with a longer operative duration. No statistically significant association between NAT and Clavien–Dindo grade ≥ III postoperative complications was detected in either the matched cohort or full‐cohort sensitivity analyses, although clinically relevant benefit or harm cannot be excluded because of the limited precision of the estimates. These findings support the technical feasibility of TaTME after NAT in experienced centres.

## Author Contributions

**Takafumi Shima:** conceptualization, methodology, investigation, formal analysis, writing – original draft. **Hiroki Hamamoto:** conceptualization, investigation, methodology, writing – review and editing, formal analysis, data curation. **Takeru Matsuda:** conceptualization, supervision, project administration, writing – review and editing. **Ichiro Takemasa:** conceptualization, supervision, project administration, writing – review and editing. **Keitaro Tanaka:** supervision, writing – review and editing. **Toshiki Mukai:** supervision, writing – review and editing. **Jun Watanabe:** supervision, writing – review and editing. **Sang‐Woong Lee:** supervision, writing – review and editing. **Seiichiro Yamamoto:** supervision, writing – review and editing. **Takeshi Naitoh:** supervision, writing – review and editing.

## Funding

The authors have nothing to report.

## Ethics Statement

The study was approved by the Institutional Review Board and Ethics Committee of Kobe University Graduate School of Medicine (IRB reference code: B220092). The need for informed consent was waived in view of the anonymity of the study data.

## Conflicts of Interest

The authors declare no conflicts of interest.

## Supporting information


**Table S1:** Sensitivity analysis of factors associated with operative time after propensity score matching.

**Table S2:** Sensitivity analyses for Clavien–Dindo grade ≥ III postoperative complications.

**Table S3:** Perioperative outcomes according to neoadjuvant treatment regimen (Regimen‐specific perioperative outcomes).

**Table S4:** Perioperative outcomes according to neoadjuvant treatment regimen (Adjusted differences in operative time (NAC as reference)).

## Data Availability

The data that support the findings of this study are available from the corresponding author upon reasonable request.

## References

[1] R. J. Heald , E. M. Husband , and R. D. H. Ryall , “The Mesorectum in Rectal Cancer Surgery—The Clue to Pelvic Recurrence?,” British Journal of Surgery 69 (1982): 613–616. 10.1002/bjs.1800691019 6751457

[2] C.‐C. Chen , Y. L. Lai , A. Y. M. Cheng , C. H. Chu , I. P. Huang , and S. H. Yang , “Transanal Total Mesorectal Excision for Rectal Cancer: Hype or New Hope?,” Journal of Gastrointestinal Oncology 10 (2019): 1193–1199. 10.21037/jgo.2019.01.13 PMC6955018 31949939

[3] Z. Wu , W. Zhou , F. Chen , W. Wang , and Y. Feng , “Short‐Term Outcomes of Transanal Versus Laparoscopic Total Mesorectal Excision: A Systematic Review and Meta‐Analysis of Cohort Studies,” Journal of Cancer 10 (2019): 341–354. 10.7150/jca.27830 PMC6360303 30719128

[4] A. Audisio , C. Gallio , V. Velenik , et al., “Total Neoadjuvant Therapy for Locally Advanced Rectal Cancer,” JAMA Oncology 11 (2025): 1045–1054. 10.1001/jamaoncol.2025.2026 PMC12246951 40638097

[5] Y. Li , C. Han , and J. Tang , “Neoadjuvant Chemoradiotherapy With or Without PD‐1 Inhibitors in MMR‐Proficient Non‐Metastatic Rectal Cancer: A Meta‐Analysis of Randomized Controlled Trials,” Frontiers in Immunology 17 (2026): 1792283. 10.3389/fimmu.2026.1792283 PMC12992012 41853263

[6] D.‐X. Zeng , R. N. Liu , X. K. Ren , et al., “Comparison of the Efficacy of Neoadjuvant Chemotherapy and Neoadjuvant Chemoradiotherapy in Locally Advanced Rectal Cancer Patients: Meta‐Analysis of Randomized Controlled Trials,” International Journal of Surgery 111 (2025): 2686–2696. 10.1097/JS9.0000000000002262 PMC12372722 39878151

[7] C. Simillis , N. Lal , S. N. Thoukididou , et al., “Open Versus Laparoscopic Versus Robotic Versus Transanal Mesorectal Excision for Rectal Cancer: A Systematic Review and Network Meta‐Analysis,” Annals of Surgery 270 (2019): 59–68. 10.1097/SLA.0000000000003227 30720507

[8] J. K. Shin , H. C. Kim , S. H. Yun , et al., “Comparison of Transanal Total Mesorectal Excision and Robotic Total Mesorectal Excision for Low Rectal Cancer After Neoadjuvant Chemoradiotherapy,” Surgical Endoscopy 35 (2021): 6998–7004. 10.1007/s00464-020-08213-z 33523275

[9] T. Matsuda , I. Takemasa , H. Endo , et al., “Local Recurrence of Rectal Cancer After Transanal Total Mesorectal Excision and Risk Factors A Nationwide Multicenter Cohort Study in Japan,” Annals of Surgery Open 1 (2024): e369. 10.1097/AS9.0000000000000369 PMC11175902 38883940

[10] E. von Elm , D. G. Altman , M. Egger , et al., “The Strengthening the Reporting of Observational Studies in Epidemiology (STROBE) Statement: Guidelines for Reporting Observational Studies,” Lancet (London, England) 370 (2007): 1453–1457. 10.1016/S0140-6736(07)61602-X 18064739

[11] D. Dindo , N. Demartines , and P.‐A. Clavien , “Classification of Surgical Complications: A New Proposal With Evaluation in a Cohort of 6336 Patients and Results of a Survey,” Annals of Surgery 240 (2004): 205–213. 10.1097/01.sla.0000133083.54934.ae PMC1360123 15273542

[12] H. Katayama , T. E. T. S. U. R. O. Tominaga , T. A. K. A. S. H. I. Nonaka , et al., “Effect of Neoadjuvant Chemotherapy in Patients With Locally Advanced Rectal Cancer: A Multicenter Propensity Score‐Matched Analysis,” Anticancer Research 42 (2022): 1527–1533. 10.21873/anticanres.15625 35220248

[13] D. Ho , K. Imai , G. King , and E. A. Stuart , “MatchIt: Nonparametric Preprocessing for Parametric Causal Inference,” Journal of Statistical Software 42 (2011): 1–28.

[14] D. Schrag , Q. Shi , M. R. Weiser , et al., “Preoperative Treatment of Locally Advanced Rectal Cancer,” New England Journal of Medicine 389 (2023): 322–334. 10.1056/NEJMoa2303269 PMC10775881 37272534

[15] P. Wu , H.‐M. Xu , and Z. Zhu , “Neoadjuvant Chemotherapy Without Radiation as a Potential Alternative Treatment for Locally Advanced Rectal Cancer: A Meta‐Analysis,” World Journal of Gastrointestinal Oncology 13 (2021): 1196–1209. 10.4251/wjgo.v13.i9.1196 PMC8465444 34616523

[16] S. Bedrikovetski , L. Traeger , W. Seow , et al., “Oncological Outcomes and Response Rate After Total Neoadjuvant Therapy for Locally Advanced Rectal Cancer: A Network Meta‐Analysis Comparing Induction vs. Consolidation Chemotherapy vs. Standard Chemoradiation,” Clinical Colorectal Cancer 23 (2024): 326–336.e9. 10.1016/j.clcc.2024.06.001 38945765

[17] G. Turri , G. Ostuzzi , G. Vita , et al., “Treatment of Locally Advanced Rectal Cancer in the Era of Total Neoadjuvant Therapy: A Systematic Review and Network Meta‐Analysis,” JAMA Network Open 7 (2024): e2414702. 10.1001/jamanetworkopen.2024.14702 PMC11151159 38833249

[18] A. Kasi , S. Abbasi , S. Handa , et al., “Total Neoadjuvant Therapy vs Standard Therapy in Locally Advanced Rectal Cancer: A Systematic Review and Meta‐Analysis,” JAMA Network Open 3 (2020): e2030097. 10.1001/jamanetworkopen.2020.30097 PMC7745099 33326026

[19] S. Liu , T. Jiang , L. Xiao , et al., “Total Neoadjuvant Therapy (TNT) Versus Standard Neoadjuvant Chemoradiotherapy for Locally Advanced Rectal Cancer: A Systematic Review and Meta‐Analysis,” Oncologist 26 (2021): e1555–e1566. 10.1002/onco.13824 PMC8417863 33987952

[20] J. B. Tuynman , H. Yao , L. R. Moolenaar , et al., “Transanal Total Mesorectal Excision Versus Laparoscopic Total Mesorectal Excision for Mid and Low Rectal Cancer (COLOR III): Short‐Term Outcomes of an International, Multicentre, Phase 3, Randomised, Controlled, Non‐Inferiority Trial,” Lancet Gastroenterology & Hepatology 11 (2026): 355–366. 10.1016/S2468-1253(26)00022-1 41887239

[21] H. Liu , Z. Zeng , H. Zhang , et al., “Morbidity, Mortality, and Pathologic Outcomes of Transanal Versus Laparoscopic Total Mesorectal Excision for Rectal Cancer Short‐Term Outcomes From a Multicenter Randomized Controlled Trial,” Annals of Surgery 277 (2023): 1–6. 10.1097/SLA.0000000000005523 PMC9762710 35815886

[22] D. K. Manatakis , N. Gouvas , J. Souglakos , and E. Xynos , “Neo‐Adjuvant Chemotherapy Alone for the Locally Advanced Rectal Cancer: A Systematic Review,” International Journal of Clinical Oncology 25 (2020): 1570–1580. 10.1007/s10147-020-01738-2 32666388

[23] Y. Wang , Y. Yang , Q.‐Q. Liu , and S.‐Z. Wang , “Compare Clinical Efficacy and Safety of Neoadjuvant Therapy and Neoadjuvant Chemoradiotherapy for Locally Advanced Rectal Cancer: Meta‐Analysis,” World Journal of Gastrointestinal Surgery 16 (2024): 1845–1856. 10.4240/wjgs.v16.i6.1845 PMC11230002 38983334

[24] Z. Xu , M. A. Valente , B. Sklow , et al., “Impact of Total Neoadjuvant Therapy on Postoperative Outcomes After Proctectomy for Rectal Cancer,” Diseases of the Colon & Rectum 66 (2023): 1022–1028. 10.1097/DCR.0000000000002555 36538720

[25] T. X. Nguyen , H. T. Ho , H. T. Phan , H. A. Vu , and N. H. Pham , “The Effectiveness of Double Team for Transanal Total Mesorectal Excision in Treatment of Mid‐Low Rectal Cancer,” International Journal of Surgery Open 34 (2021): 100359.

